# Computational chemistry in pharmaceutical research – where do we stand after 25 years?

**DOI:** 10.1186/1758-2946-4-S1-O19

**Published:** 2012-05-01

**Authors:** Herbert Köppen

**Affiliations:** 1 Schützenpfad 27, 55218 Ingelheim/D, Germany

## 

In the early 80^th^ of the last century molecular modeling was introduced as a tool to aid rational drug design in pharmaceutical industry. However in these early days modeling had narrow limits due to the lack of adequate compute power and sophisticated methods. There was a large gap between expectations of medicinal chemists and the power of theoretical methods. Today computational chemistry is an integral part of pharmaceutical research. What has changed over the last decades, what was the driving force of the progress?

The talk is based on the author’s experience at Boehringer Ingelheim and will focus on computational chemistry methods with proven value in industrial pharmaceutical research. Amongst others the synergy of virtual screening and combinatorial chemistry and the impact of the recently published structures of GPCRs on pharmaceutical research will be illustrated. Further current challenges, e.g. the consideration of protein flexibility, will be discussed.

